# Costs associated with febrile neutropenia in solid tumor and lymphoma patients – an observational study in Singapore

**DOI:** 10.1186/1472-6963-14-434

**Published:** 2014-09-24

**Authors:** Xiao Jun Wang, Mabel Wong, Li Yang Hsu, Alexandre Chan

**Affiliations:** Department of Pharmacy, Faculty of Science, National University of Singapore, Block S4A, Level 3, 18 Science Drive 4, Singapore, 117543 Singapore; Department of Pharmacy, National Cancer Centre Singapore, Singapore, Singapore; Department of Medical Oncology, National Cancer Centre Singapore, Singapore, Singapore; Department of Medicine, National University Health System Singapore, Singapore, Singapore

**Keywords:** Febrile neutropenia, Neutropenia, Fever, Cost analysis, Factors

## Abstract

**Background:**

The primary objective was to describe the total direct inpatient costs among solid tumor and lymphoma patients with chemotherapy-induced febrile neutropenia (FN) and the factors that were associated with higher direct cost. The secondary objective was to describe the out-of-pocket patient payments and the factors that were associated with higher out-of-pocket patient payments.

**Methods:**

This was a single-center observational study conducted at the largest cancer center in Singapore. All of the adult cancer patients hospitalized due to FN from 2009 to 2012 were studied. The primary outcomes were the total hospital cost and the out-of-pocket patient payments (adjusted by government subsidy) per FN episode. Univariate analysis and multiple linear regression were conducted to identify the factors associated with higher FN costs.

**Results:**

Three hundred and sixty seven adult cancer patients were documented with FN-related hospitalizations. The mean total hospital cost was US$4,193 (95% CI: US$3,779-4,607) and the mean out-of-pocket patient payment was US$2,230 (95% CI: US$1,976-2,484), per FN episode. The factors associated with a higher total hospital cost were longer length of stay, severe sepsis, and lymphoma as underlying cancer. The out-of-pocket patient payment was positively associated with longer length of stay, severe sepsis, lymphoma diagnosed as underlying cancer, the therapeutic use of granulocyte colony-stimulating factor (GCSF), the private ward class, and younger patients.

**Conclusions:**

The total hospital cost and out-of-pocket patient payments of FN management in lymphoma cases were substantial compared with other solid tumors. Factors associated with a higher FN management cost may be useful for developing appropriate strategies to reduce the cost of FN for cancer patients.

## Background

Febrile neutropenia (FN) is a common complication in cancer patients receiving myelosuppressive chemotherapy. A recent study conducted by our research group [1] reported that even if cancer patients were given prophylactic myeloid growth factor support, 16.6% of the patients suffered at least one breakthrough FN episode during their chemotherapy. FN is also potentially life-threatening. Inpatient mortality rates of between 4.7% and 9.5% were recently reported [2–4]. FN often leads to a chemotherapy dose reduction and to treatment delays, which may affect the patients’ long-term clinical outcomes [3, 5–7]. A previous study [8] indicated that patients who received a reduced chemotherapy intensity (relative dose intensity ≤ 90%) achieved significantly less survival years than patients who received the optimal chemotherapy dose intensity.

Aside from its clinical consequences, FN also has a substantial economic effect, particularly in the inpatient setting [9]. Three U.S. studies [3, 10, 11] estimated that the average costs of FN inpatient management ranged from US$18,880 to US$22,086. The direct costs for outpatient management were considerably lower than inpatient care, at US$985 per episode [11]. Similar trends with a different cost burden degree were observed in Europe. A German prospective study [12] analyzed the influence of FN on the use of health resources and costs for patients with both solid cancers and lymphoma and found that the estimated mean direct cost per FN episode requiring hospital care was €3,950. A Spanish study [13] found a mean direct cost per episode attributable to FN of €3,841 in a similar patient population. In a recent study conducted in Ireland [14], the mean cost per FN episode in the inpatient setting was estimated to be €8,915. The cost of FN therefore varies greatly across different countries and health care systems.

In Singapore, limited data have been reported on the cost of the management of FN on cancer patients. Cost studies are therefore needed to make accurate estimates of the cost of FN on patients with various types of cancers. This knowledge can be used to develop further economic evaluations of the current FN management strategies, which may help in clinical decision-making. Therefore, the primary objective of this study was to describe the total direct inpatient costs among the solid tumor and lymphoma patients who received inpatient management of chemotherapy-induced febrile neutropenia (FN) and the factors that were associated with higher direct cost. The secondary objective was to describe the out-of-pocket patient payments and the factors that were associated with the higher out-of-pocket patient payments. We expected this study to provide an accurate estimate of the cost of FN, reflecting the local management in Singapore. This study is a fundamental requirement for the development of further economic evaluations of the current FN management strategies and for reducing the cost of FN on cancer patients in Singapore

## Methods

### Study design and setting

This was a secondary analysis of a prospective study [15] conducted at the National Cancer Centre Singapore (NCCS) in 2014. In the original study, the clinical efficacy of the adjunctive G-CSF among cancer patients with febrile neutropenia was evaluated. Clinical data (demographics, medical history, medication history, and treatment outcomes) were prospectively obtained from patients as well as hospital information support system and the pharmacy prescription database. The complete methods for the data collection are described in detail elsewhere [15]. The study was approved by the SingHealth Institutional Review Board. The NCCS is an ambulatory cancer center that treats approximately 70% of all of the solid tumor and lymphoma cases in Singapore [16]. At the NCCS, the majority of the patients who develop FN are hospitalized for treatment until they recover from their low absolute neutrophil count (ANC) and fever [17]. The inpatient management of FN follows the guidelines from the Infectious Diseases Society of America (IDSA) [18].

In Singapore, inpatient care is classified into private ward (Class A and Class B1) and subsidized ward (Class B2 and Class C) patients. The Class A (single bed) ward is not subsidized by the government. Different levels of government subsidy are available for the Class B1 (four beds), Class B2 (six beds), and Class C (open ward) wards for Singapore citizens and permanent residents [19, 20]. The government subsidizes 0% to 20% of the total hospital bill for Singapore citizens and permanent residents in the Class B1 wards. In contrast, citizens and permanent residents in the Class B2 and Class C wards receive subsidy of up to 80% of their total bill [20]. The Goods and Services Tax is absorbed for those who are eligible for subsided treatment, related services and/or who are staying in the Class B2 and C wards [19].

### Patient population

All of the adult cancer patients (aged 18 years and above) who received chemotherapy for the treatment of solid tumors or lymphoma at the NCCS and were hospitalized for FN between 2009 and 2012 were included in this study. The definition of FN was based on the IDSA guidelines [18]. The cancer type was determined using the ninth revision of the International Classification of Diseases – Clinical Modification (ICD-9-CM). Patients who were hospitalized more than two days before their FN episode were excluded. If a patient required 10 days or more of hospitalization after recovering from fever and low ANC, the patient was also excluded from this analysis. In patients who had experienced multiple FN-related hospitalizations during the study period, the first hospitalization was selected for analysis.

### Cost data

The cost data were estimated from billing data obtained via the financial electronic databases of the NCCS and the Singapore General Hospital, which included the total hospital cost, the reimbursement bill, the out-of-pocket patient payments, and the cost breakdown of out-of-pocket patient payments (consisted of ward charges, laboratory charges, radiology charges, prescription charges, surgical charges, and other charges). A hospital’s perspective was taken in this study. The FN-related costs included all of the costs incurred from the day of hospitalization until the discharge date. These medical costs were expressed as the total hospital cost and the out-of-pocket patient payments (adjusted by the government subsidy). The resource and costs associated with the treatment of the underlying cancer were not included. All of the cost data were adjusted to 2012 Singapore dollars (SG$) using the health care component of the Singapore Consumer Price Index [21]. The cost data were then converted to 2012 U.S. dollars (US$) using purchasing power parity (PPP) conversion rate obtained from the World Bank (2012; $SG1 = $US0.7157). The out-of-pocket patient payments was broken down into its individual cost components[1]. The information on disaggregated cost of total hospital cost was not available.

### Study outcomes and definitions

The primary outcomes of this analysis were the total hospital cost and the out-of-pocket patient payments per FN episode. Univariate and multivariate analyses were performed to identify the clinical factors which had significant associations with a higher total hospital cost or out-of-pocket patient payments per FN episode. We evaluated the patient demographics (age, gender, and ethnicity), major cancer diagnoses (breast cancer, lymphoma, gastrointestinal cancer, head and neck cancer, lung cancer, sarcoma, genitourinary cancer, gynecologic cancer, and others), length of stay, treatment with therapeutic GCSF, admission source (emergency department, clinic, and inpatient referral), Multinational Association of Supportive Care in Cancer (MASCC) score, ward class (Class A, Class B1, Class B2, and Class C), severity of sepsis, duration of antibiotics, and the ANC at presentation and nadir. The MASCC score is used to identify low-risk patients for serious complications of febrile neutropenia. The patient with higher MASCC score (score ≥21 points) possesses a lower risk to develop serious complications from FN [22].

### Statistical analysis

The clinical and demographic characteristics were reported for each patient based on a single admission per patient. We evaluated all of the admissions associated with FN. Descriptive analyses were used to summarize the primary study outcomes of this study (total hospital cost and out-of-pocket patient payments). The means, median, and 95% confidence intervals (CI) were reported. For the univariate and multivariate analyses, the total hospital cost and the out-of-pocket patient payments per FN episode were transformed on the natural logarithmic scale because an exploratory analysis revealed that the two dependent variables were not normally distributed. Group comparisons of parametric data (natural logarithmic transformed cost data) were conducted using Student t-test or analysis of variance (ANOVA). Scheffe’s Test with Bonferroni correction was performed if there was a statistically significant finding through the ANOVA test. This step was performed to identify the variables of interest which may affect the outcomes (total hospital cost and out-of-pocket patient payments) in this study. Pearson’s correlation coefficient analysis was conducted to identify the variables significantly associated with a higher total hospital cost or out-of-pocket patient payments. After identifying the variables which were significantly associated with higher costs, multiple linear regression models were conducted to estimate the total hospital cost and out-of-pocket patient payments. IBM SPSS Statistics 21.0 was used for all of the statistical analysis.

## Results

### Patient population

Three hundred and sixty seven adult cancer patients were documented with FN-related hospitalizations between 2009 and 2012. The average patient age was 54.8 years (range, 18–79), 37.9% of the patients were male, and 77.1% were of Chinese ethnicity. The most common primary cancer types were breast cancer (36.8%), followed by lymphoma (21.5%), gastrointestinal cancer (10.6%), head and neck cancer (9.3%), and lung cancer (8.4%) (Table 1). GCSF was used as a therapeutic treatment (86.9%) in the majority of the FN episodes. Inpatient care commonly occurred (78.7%) in the subsidized wards (Class B2, Class C) (Table 2). The majority of the patients (90.5%) were Singapore citizens or permanent residents.

**Table 1:** **Clinical and demographic characteristics of the subjects**

Baseline characteristics	Number of patients (n = 367)
**Age in years**	
Mean (SD)	54.8 (12.2)
18-40 (%)	41 (11.2%)
41-60 (%)	202 (55.0%)
61-80 (%)	124 (33.8%)
**Gender**	
Male (%)	139 (37.9%)
Female (%)	228 (62.1%)
**Ethnicity**	
Chinese (%)	283 (77.1%)
Malay (%)	38 (10.4%)
Indian (%)	20 (5.4%)
Other (%)	26 (7.1%)
**Cancer Type**	
Breast CA (%)	135 (36.8%)
Lymphoma (%)	79 (21.5%)
Gastrointestinal CA (%)	39 (10.6%)
Head and Neck CA (%)	34 (9.3%)
Lung CA (%)	31 (8.5%)
Sarcoma (%)	16 (4.4%)
Genitourinary CA (%)	15 (4.1%)
Gynecologic CA (%)	13 (3.5%)
Other/Unknown CA (%)	5 (1.4%)
**MASCC Risk**	
Low (%)	300 (81.7%)
High (%)	67 (18.3%)
**Outcome**	
Death (%)	14 (3.8%)
Recover (%)	353 (96.2%)

**SD** = standard deviation; **CA** = cancer; **MASCC** = Multinational Association of Supportive Care in Cancer.

**Table 2:** **Descriptive analysis of the total hospital cost and the out-of-pocket patient payments (n = 367)**

		Total hospital cost			Out-of-pocket patient payments		
Variables	No. (%)	per episode, 2012 US$			per episode, 2012 US$		
		Mean (±95% CI)	p value	Median	Mean (±95% CI)	p value	Median
**All subjects**	367 (100%)	4,193 (±414)		2,837	2,230 (±254)		1,405
**Age in years**							
18-40	41 (11.2%)	5,160 (±1,791)	0.182 ^@^	3,456	3,031 (±730)	0.001 ^@^	2,468
41-60	202 (55.0%)	4,089 (±544)		2,723	2,105 (±303)		1,337
61-80	124 (33.8%)	4,044 (±624)		3,022	2,167 (±514)		1,369
**Gender**							
Male	139 (37.9%)	5,286 (±864)	< 0.001 ^#^	3,432	2,795 (±558)	0.006 ^#^	1,510
Female	228 (62.1%)	3,527 (±389)		2,651	1,885 (±218)		1,334
**Cancer type**							
Breast CA	135 (36.8%)	2,898 (±369)	< 0.001 ^@^	2,208	1,567 (±249)	< 0.001 ^@^	1,167
Lymphoma	79 (21.5%)	6,560 (±1,362)		4,159	3,389 (±827)		1,972
Gastrointestinal CA	39 (10.6%)	4,145 (±859)		3,432	1,927 (±541)		1,349
Head and Neck CA	34 (9.3%)	4,565 (±1,665)		3,150	2,502 (±867)		1,486
Lung CA	31 (8.4%)	3,353 (±912)		2,314	1,861 (±947)		1,097
Sarcoma	16 (4.4%)	3,857 (±1,119)		3,479	2,331 (±701)		2,194
Genitourinary CA	15 (4.1%)	4,898 (±2,803)		2,654	2,629 (±1,498)		1,340
Gynecologic CA	13 (3.5%)	4,615 (±1,482)		3,858	2,877 (±1,317)		2,251
Other/Unknown CA	5 (1.4%)	2,707 (±1,199)		2,359	1,403 (±677)		1,364
**Therapeutic GCSF**							
No	48 (13.1%)	3,752 (±1,088)	0.064 ^#^	2,392	1,544 (±384)	0.007 ^#^	1,181
Yes	319 (86.9%)	4,267 (±450)		2,950	2,338 (±286)		1,487
**Admission source**							
ED	195 (53.1%)	3,721 (±503)	< 0.001 ^@^	2,577	1,979 (±259)	0.001 ^@^	1,302
NCCS clinic	121 (33.0%)	3,671 (±594)		2,451	2,069 (±409)		1,377
Inpatient referral	51 (13.9%)	7,238 (±1,601)		4,436	3,570 (±1,163)		2,112
**Severe sepsis**							
No	315 (85.8%)	3,590 (±350)	< 0.001 ^#^	2,571	2,044 (±264)	< 0.001 ^#^	1,283
Yes	52 (14.2%)	7,879 (±1,744)		6,105	3,358 (±772)		2,073
**MASCC risk**							
Low	300 (81.7%)	3,790 (±412)	< 0.001 ^#^	2,571	2,162 (±283)	0.034 ^#^	1,316
High	67 (18.3%)	5,998 (±1,249)		4,436	2,533 (±574)		1,711
**Ward class**							
Class A1	30 (8.2%)	5,879 (±1,875)	0.039 ^@^	4,086	5,245 (±1,889)	< 0.001 ^@^	3,828
Class B1	48 (13.1%)	3,699 (±784)		2,479	3,749 (±835)		2,512
Class B2	194 (52.9%)	3,855 (±540)		2,727	1,696 (±205)		1,256
Class C	95 (25.8%)	4,602 (±923)		3,185	1,600 (±313)		1,100

**ED** = emergency department; **NCCS** = National Cancer Centre Singapore; **CI** = confidence interval; **MASCC** = Multinational Association of Supportive Care in Cancer; **CA** = cancer; ^**@**^p value from ANOVA test; ^**#**^p value from two sample t-test, assumed equal variance

### Total hospital cost

The mean and median total hospital cost per FN episode for all of the hospitalizations were US$4,193 (95% CI: US$3,779-4,607) and US$2,837, respectively. A subgroup analysis by cancer type showed that the patients with lymphoma had the highest mean total hospital cost of US$6, 560 (95% CI: US$5,198-7,922), followed by patients with genitourinary cancer (US$4,898, 95% CI: US$2,095-7,701), and gynecologic cancer (US$4, 615, 95% CI: US$3,133-6,097). Breast cancer patients had the lowest mean total hospital cost (US$2, 898, 95% CI: US$2,529-3,267) of the identified cancer types (Table 2).

FN episodes that manifested with severe sepsis had a considerably higher mean cost (US$7,879, 95% CI: US$6, 135-9,623) than those without sepsis (US$3,590, 95% CI: US$3,240-3,940). Other factors associated with a higher mean total hospital cost were inpatient referrals (US$7,238, 95%CI: US$5,637-8,839) and a low MASCC score (US$5,998, 95% CI: US$4,749-7,247).

### Out-of-pocket patient payments

The mean and median out-of-pocket patient payments per FN episode were US$2,230 (95% CI: US$1,976-2,484) and US$1,405, respectively. A subgroup analysis by cancer type revealed that the patients with lymphoma had the highest mean out-of-pocket patient payments of US$3,389 (95% CI: US$2,562-4,216), followed by patients with gynecologic cancer (US$2,877, 95% CI: US$1,560-4,194), and genitourinary cancer (US$2,629, 95% CI: US$1,131-4,127). Breast cancer patients had the lowest mean out-of-pocket patient payments (US$1,567, 95% CI: US$1,318-1,816) of the known cancer types (Table 2).

The mean out-of-pocket patient payments of FN episodes with severe sepsis (US$3,358, 95% CI: US$2,586-4,130) was higher than without sepsis (US$2,044, 95% CI: US$1,780-2,308). Higher FN out-of-pocket patient payments were associated with the use of therapeutic GCSF (US$2,338, 95% CI: US$2,052-2,624) and the private wards (Class A (US$5,245, 95% CI: US$3,356-7,134) and Class B1 (US$3,749, 95% CI: US$2,914-4,584)). The main cost component identified in the out-of-pocket patient payments was the prescription charges (35.2%), followed by the ward charges (18.4%) and the laboratory cost (13.3%). Approximately one quarter of the total out-of-pocket patient payments was unclassified (29.4%) (Table 3).

**Table 3:** **Cost breakdown of the total out-of-pocket patient payments (n=367)**

	All of the febrile neutropenia episodes		
	Median cost (interquartile range), 2012 US$	Mean cost (95% CI), 2012 US$	Percentage breakdown
Ward	230 (145–504)	432 (375–488)	18.4%
Laboratory	171 (120–260)	314 (266–362)	13.3%
Radiology	17 (8–64)	88 (66–110)	3.7%
Prescription	507 (319–891)	829 (720–937)	35.2%
Other/Unknown	383 (226–661)	692 (585–800)	29.4%
Total	1,405 (901–2,442)	2,230 (1,976-2,483)	100%

### Univariate analysis

The association of cancer types with the total hospital cost was evaluated. Patients with lymphoma had a significantly higher cost (p < 0.001) than patients with other cancer types. Male patients had a significantly higher total hospital cost (p < 0.001) than female patients. Other factors associated with a significantly higher total hospital cost were inpatient referrals (p < 0.001), severe sepsis (p < 0.001), and a low MASCC score (p < 0.001) (Tables 2 and 4). In the correlation analysis, a higher total hospital cost was also significantly associated with a longer length of stay (r = 0.86, p < 0.01), a lower ANC at presentation (r = 0.17, p < 0.01), and a longer time to recover the ANC (r = 0.16, p < 0.01).

**Table 4:** **Comparisons of costs among subsets of variables identified in univariate analysis (n=367)**

Variables	Reference (r)	Comparator (c)	Out-of-pocket patient payments, 2012 US$		Total hospital cost, 2012 US$	
			Mean difference (r-c)	p value ^@^	Mean difference (r-c)	p value ^@^
Cancer type	Lymphoma	Breast CA	1,822	< 0.001*	3,662	< 0.001*
		Gastrointestinal CA	1,462	0.411	2,415	0.634
		Head & Neck CA	887	0.999	1,995	0.94
		Lung CA	1,528	0.098	3,207	0.072
		Sarcoma	1,058	1	2,703	0.988
		Genitourinary CA	760	0.952	1,662	0.804
		Gynecologic CA	512	1	1,945	1
		Other/Unknown CA	1,986	0.969	3,853	0.87
Admission source	Inpatient referral	NCCS clinic	1,501	0.002*	3,567	< 0.001*
		ED admission	1,591	0.003*	3,517	< 0.001*
Age in years	18-40 yrs	41-60 yrs	926	0.002*		
		61-80 yrs	864	0.005*		
Ward class	A1	B1	1,496	0.763		
		B2	3,549	< 0.001*		
		C	3,645	< 0.001*		
	B1	A1	-1,496	0.763		
		B2	2,053	< 0.001*		
		C	2,149	< 0.001*		

**ED** = emergency department; **NCCS** = National Cancer Centre Singapore; **CA** = cancer; * significant at α = 0.05 after Bonferroni correction; ^@^ Conducted by using Scheffe’s Test with Bonferroni correction.

The association of cancer types with the out-of-pocket patient payments was also evaluated. The lymphoma group again had a significant higher cost (p < 0.001) than the other cancer types. The patients in the 18–40 years age group (p < 0.01) and male patients (p < 0.01) also had a significantly higher cost than older patients and female patients, respectively. Other factors associated with higher out-of-pocket patient payments were treatment with therapeutic GCSF (p < 0.01), inpatient referrals (p < 0.01), severe sepsis (p < 0.001), and a lower MASCC score (p < 0.05). Patients staying in private wards (Class A/B1) were associated with a significantly higher out-of-pocket patient payments (p < 0.001) than those in the subsided wards (Class B2/C) (Tables 2 and 4). The correlation analysis revealed that the out-of-pocket patient payments was positively correlated with a longer length of stay (r = 0.63, p < 0.01), a lower ANC at presentation (r = 0.11, p < 0.05), and a longer time to recover the ANC (r = 0.127, p < 0.05).

### Multivariate analysis

Table 5 shows the factors that were found to be significantly associated with a higher total hospital cost. The factors were the length of stay (coefficient = 0.061, 95% CI: 0.056 ~ 0.065, p < 0.001), lymphoma (coefficient = 0.105, 95% CI: 0.064-0.146, p < 0.001), and severe sepsis (coefficient = -0.131, 95% CI: 0.086-0.176, p < 0.001). This multivariate model for the total hospital cost had an adjusted R^2^ of 0.782 (p < 0.001) (Table 5).

**Table 5:** **Factors associated with the total hospital cost in a regression model (n=367)** ^**a**^

Variables ^b^	Unstandardized coefficient (B)	95% CI for B		Standard error	Standardized coefficient (B)	p value ^c^
		Lower bound	Upper bound			
Length of stay	0.061	0.056	0.065	0.002	0.763	**< 0.001**
Time to ANC recovery	0.003	-0.003	0.009	0.003	0.024	0.363
ANC at presentation	0.008	-0.054	0.070	0.031	0.007	0.797
MASCC score < 21	-0.013	-0.052	0.026	0.020	-0.017	0.513
Lymphoma	0.105	0.064	0.146	0.021	0.141	**< 0.001**
Male gender	0.026	-0.006	0.059	0.016	0.042	0.110
Severe sepsis	0.131	0.086	0.176	0.023	0.154	**< 0.001**
Inpatient referral	0.038	-0.008	0.083	0.023	0.043	0.107

**a.** Adjusted R^2^ = 0.782, p < 0.001; **b.** Only clinically relevant variables that were statistically significant at p value of 0.05 in the univariate analysis were included in the regression model; **c.** Bolded p values are statistically significant; **ANC** = absolute neutrophil count; **MASCC** = Multinational Association of Supportive Care in Cancer.

A separate multivariate model was used to identify the significant predictors for higher out-of-pocket patient payments, which are shown in Table 6. The factors were the length of stay (coefficient = 0.054, 95% CI: 0.049-0.060, p < 0.001), lymphoma (coefficient = 0.067, 95% CI: 0.012-0.121, p = 0.017), severe sepsis (coefficient = 0.081, 95% CI: 0.020-0.142, p = 0.009), 18–40 years age group (coefficient = 0.082, 95% CI: 0.019-0.144, p = 0.011), treatment with therapeutic GCSF (coefficient = 0.101, 95% CI: 0.041-0.161, p = 0.001), and staying on a private ward (coefficient = 0.408, 95% CI: 0.359-0.457, p < 0.001). The regression model had an adjusted R^2^ of 0.691 (Table 6).

**Table 6:** **Factors associated with higher out-of-pocket patient payments in a regression model (n=367)** ^**a**^

Variables ^b^	Unstandardized coefficient (B)	95% CI for B		Standard error	Standardized coefficient (B)	p value ^c^
		Lower bound	Upper bound			
Length of stay	0.054	0.049	0.060	0.003	0.614	**< 0.001**
Time to ANC recovery	0.004	-0.004	0.013	0.004	0.031	0.320
ANC at presentation	-0.003	-0.085	0.080	0.042	-0.002	0.948
MASCC score < 21	0.019	-0.033	0.072	0.027	0.022	0.473
Lymphoma	0.067	0.012	0.121	0.028	0.080	**0.017**
Male gender	0.017	-0.027	0.060	0.022	0.024	0.452
Severe sepsis	0.081	0.020	0.142	0.031	0.085	**0.009**
Inpatient referral	0.013	-0.048	0.074	0.031	0.013	0.683
18-40 year age group	0.082	0.019	0.144	0.032	0.078	**0.011**
Therapeutic GCSF treatment	0.101	0.041	0.161	0.030	0.100	**0.001**
Private Ward (Class A or B1)	0.408	0.359	0.457	0.025	0.499	**< 0.001**

**a.** Adjusted R^2^ = 0.691, p < 0.001; **b.** Only clinically relevant variables that were statistically significant at p value of 0.05 in the univariate analysis were included in the regression model; **c.** Bolded p values are statistically significant; **ANC** = absolute neutrophil count; **GCSF** = granulocyte colony-stimulating factor; **MASCC** = Multinational Association of Supportive Care in Cancer.

## Discussion

In this study, we described the total hospital cost and out-of-pocket patient payments among solid tumor and lymphoma patients who received inpatient management of chemotherapy-induced FN in Singapore. The factors associated with the total hospital cost and out-of-pocket patient payments were also identified. The factors that were found to be associated with a higher total hospital cost for FN treatment were a longer length of stay, severe sepsis, and lymphoma. The factors associated with a higher out-of-pocket patient payments were a longer length of stay, severe sepsis, lymphoma, the age group 18–40 years, treatment with therapeutic GCSF during admission, and staying on a private ward (Class A or B1).

When analyzed by cancer type, the mean total hospital cost was US$6,560 for lymphoma patients, US$2,898 for breast cancer patients, and ranged from US$3,353 to US$4,898 for patients with other identified cancers. A similar pattern was found in previous studies [11, 12, 23]. Among cancer patients who received inpatient care, the Bennett [23] study estimated that the mean direct cost was US$21,601 for lymphoma patients, which was much more costly than that of breast cancer (US$13,186) or other cancers (US$12,150). A recently published Spanish retrospective study [13] reported that the cost was highest when lymphoma was the underlying cancer (€4,514), followed by breast cancer (€3,518) and lung cancer (€3,310). This difference was probably related to a longer length of stay due to lymphoma patients suffering FN episodes [10]. One study showed that lymphoma patients had the longest length of stay (10.1 days) and patients with female breast cancer had the shortest mean length of stay (5.9 days) [10]. The length of stay result was consistent with the cost, as lymphoma had the highest mean total hospital cost (US$24,218) and female breast cancer had the lowest mean cost (US$11,132). As the length of stay was directly associated with the total hospital cost, interventions to improve FN management in future should focus on decreasing the duration of the hospital stay.

A lower out-of-pocket patient payment was associated with the Class B2 and C wards. The majority of the patients were citizens or residents and they received more subsidies in these wards than in the other ward classes. The government only subsidizes 0% to 20% of the total hospital bill for Singapore citizens and permanent residents in the private Class A and Class B wards [20]. In contrast, citizens and permanent residents in the Class B2 and Class C wards can receive a subsidy of up to 80% of their total bill [20]. As the patients’ choice of ward class may reflect their preference or values as well as their socioeconomic status, which are an important determinant in clinical decision-making, future cost-effective analysis or cost-utility analysis studies should collect the willingness to pay value separately, based on the different ward classes.

Prescription charges were the largest component of the overall out-of-pocket patient payments of FN, followed by the ward charges, and the laboratory cost. The medication costs and ward costs were therefore the main cost drivers in the inpatient management of FN. A micro-costing method can be used to account for the cost of the ward and medication, if a more accurate estimate of the direct medical cost of FN is required. Prescription charges were identified as the largest component in the out-of-pocket patient payments because the reimbursement of healthcare resources in Singapore differs from other countries. The government subsidy for cancer patients mainly focuses on the hospitalization charges and the chemotherapy charges [24]. Certain costly supportive care medications that are frequently used in the management of FN, such as G-CSF, are not fully covered for reimbursement. Therefore, cost-effective analysis on those medications may be useful to formulate the strategies to reduce patients’ out-of-pocket payments. In addition, it has been demonstrated that medication errors (such as inappropriate medication dosing) in the management of infectious diseases may contribute to higher medication costs among patients [25]. Future studies should aim to lower the cost burden of FN by focusing on interventions that can improve medication management, such as having a clinical pharmacist to review prescribed medications to reduce potential medication errors, and to optimize the medication management strategies [25].

There are a number of limitations to this study. A large proportion of the expenses in the total out-of-pocket patient payments could not be classified. This may introduce some variability in identifying the main cost drivers of the cost of FN. We did not capture patients’ comorbidities in detail, hence we were unable to analyze the contribution of comorbidities to the cost burden of FN. Our inclusion criteria were stricter than other studies, which makes comparisons of the cost estimates difficult [1, 3, 10, 26]. The cost was calculated from the start date of an FN episode until the discharge date. Additional costs due to comorbidity or cancer-related morbidity and mortality were not considered. Those patients whose discharge date was more than 10 days after both their fever and ANC recovered were excluded from the analysis. These criteria were applied so that we only studied the cost of the management of FN, not the other costs that are associated with the management of comorbidities or cancer due to an extended hospital stay. Due to the unavailability of data breakdown, micro-costing on the total hospital cost was not feasible. However, the average cost-per-case estimates were used to evaluate the total hospital cost of FN in this study.

## Conclusions

In conclusion, the total hospital cost and out-of-pocket patient payments of FN management in lymphoma cases were substantially greater than in other solid tumor cases. A longer length of stay and severe sepsis were associated with a higher total hospital cost for FN management. The factors associated with a higher financial burden on patients with FN were also identified. These results may be useful for further economic evaluation to develop appropriate strategies to reduce the cost of FN on cancer patients.
